# Bacteriophages and Their Host Range in Multidrug-Resistant Bacterial Disease Treatment

**DOI:** 10.3390/ph16101467

**Published:** 2023-10-16

**Authors:** Ka Mun Chung, Xiew Leng Liau, Swee Seong Tang

**Affiliations:** 1 Division of Microbiology and Molecular Genetics, Institute of Biological Sciences, Faculty of Sciences, University Malaya, Kuala Lumpur 50603, Malaysia; 2 Centre for Research in Biotechnology for Agriculture, University Malaya, Kuala Lumpur 50603, Malaysia

**Keywords:** multidrug-resistant (MDR) bacteria, phage host range, narrow host range, broad host range, polyvalent, monovalent

## Abstract

The rapid emergence of multidrug-resistant (MDR) bacteria in recent times has prompted the search for new and more potent antibiotics. Bacteriophages (commonly known as phages) are viruses that target and infect their bacterial hosts. As such, they are also a potential alternative to antibiotics. These phages can be broadly categorized into monovalent (with a narrow host range spectrum and specific to a single bacterial genus) and polyvalent (with a broad host range and specific to more than two genera). However, there is still much ambiguity in the use of these terms, with researchers often describing their phages differently. There is considerable research on the use of both narrow- and broad-host range phages in the treatment of infections and diseases caused by MDR bacteria, including tuberculosis, cystic fibrosis, and carbapenem-resistant *Enterobacterales* (CRE) infectious diseases. From this, it is clear that the host range of these phages plays a vital role in determining the effectiveness of any phage therapy, and this factor is usually analyzed based on the advantages and limitations of different host ranges. There have also been efforts to expand phage host ranges via phage cocktail development, phage engineering and combination therapies, in line with current technological advancements. This literature review aims to provide a more in-depth understanding of the role of phage host ranges in the effectiveness of treating MDR-bacterial diseases, by exploring the following: phage biology, the importance of phages in MDR bacteria diseases treatment, the importance of phage host range and its advantages and limitations, current findings and recent developments, and finally, possible future directions for wide host range phages.

## 1. Introduction

Multidrug resistance (MDR) has become a growing public health concern globally as more microbes, including bacteria, have developed new resistance mechanisms to counter the antibiotics and chemotherapeutic agents designed to kill them. Over the past decade, the number of MDR organisms is reported to have increased dramatically. Moreover, antibiotics reportedly no longer work effectively on several species of common bacteria. These are termed ESKAPE pathogens, an acronym that encompasses *Enterococcus faecium*, *Staphylococcus aureus*, *Klebsiella pneumoniae*, *Acinetobacter baumannii*, *Pseudomonas aeruginosa*, and *Enterobacter faecium* [1]. Notably, mechanisms such as horizontal resistance gene transfer and multidrug efflux pump systems that pump antibiotics out of the cell can both enable acquired resistance in MDR organisms [2]. This development is associated with prolonged illnesses, increased medical costs, high mortality rates and foodborne disease outbreaks, making MDR a global interest and high-priority issue [3]. According to the World Health Organization (WHO), the main causes of antimicrobial resistance include the misuse or overuse of antibiotics; inadequate prevention and control; a lack of clean water, proper sanitation and hygiene; a lack of awareness and education; and finally, the inadequate enforcement of legislation [4]. 

The increasing threat posed by these MDR bacteria has prompted the re-emergence of bacteriophages (viruses that infect and kill bacteria specifically) as a potential alternative to antibiotic regimens [5]. In fact, the first recorded successful clinical use of bacteriophage therapy via intravenous route was conducted only quite recently, in 2016, at the University of California San Diego (United States, U.S.), where a phage was used to treat a severe MDR *A. baumannii* infection. Phage treatment has sparked increased public interest ever since that milestone. Phage banks and international phage directories have also been gradually developed to facilitate the conversion of phage research into clinical use [6]. As of now, phage therapy has been given emergency use authorization (EUA) by the U.S. Food and Drug Administration (FDA), which permits the compassionate use of this therapy depending on the severity of the patients’ cases. Patients with extensive MDR infections, such as cystic fibrosis, with underlying pulmonary infections where antibiotics do not work effectively, are often granted compassionate use [7]. 

However, commercializing bacteriophages has proven a challenge worldwide due to the absence of a gold standard in phage preparation and administration, as well as difficulties in conducting clinical trials and applying the results of these. There remain, moreover, further gaps in knowledge in this area of study, such as in the effectiveness, host range and safety of phage usage. Currently, the U.S. and China are the most active countries in phage therapy research [6]. While continents such as South America and Asia contain a large potential biological reservoir (flora and fauna) where novel phages could be isolated, funds for phage research have been limited in these areas. 

The efficiency and safety of using phage therapy to tackle MDR bacteria have been under study for many years. The key advantage of using phage therapy over antibiotics is that phages infect and kill specific bacteria without destroying other normal flora that exist in the body. In addition to therapeutic agents, phages have also been widely used as detection biomarkers [8] and biological controls [9] for pathogenic bacteria in various sectors. In addition, the interactions between phages and their host cells have been extensively studied to obtain a more in-depth understanding of the specificity of phages. 

A key factor in the use of phages is that most can only infect and kill the bacteria carrying their complementary receptor. This effectively determines the host range of lytic phages. It is also important to understand how the host range of phages affects their effectiveness in the treatment of MDR bacterial diseases. Based on their host range spectrum, phages can be generally categorized as either monovalent or polyvalent. Polyvalent phages have a wide host range and are specific to more than two genera of bacteria. Monovalent phages, in contrast, work only with a limited host range spectrum [10]. 

Current research on phage treatment in MDR bacterial diseases has highlighted the advantages and limitations of both broad and narrow host ranges. Most documented phages fall into the latter category, showing only limited specificity because they are species- or strain-specific. It was initially thought that having a phage with a limited host range—one that only infects one species—would stop the phage virus from eradicating the other bacteria that make up the human microbiome. However, a broad-host range phage that kills several different bacterial species is similar to a broad-spectrum antibiotic, making it beneficial for phage therapy in treating bacterial co-infections. Indeed, phages are nowadays usually devised as a cocktail, primarily to avoid the emergence of phage resistance and to increase the effectiveness of phage treatment. Phage cocktails can be devised on a case-by-case basis. Cocktails that exhibit a narrow host range can be used to combat phage resistance in specific infectious bacterium by infecting the same type of bacterium but using different receptors. On the other hand, broad-host range phage cocktails are useful for infections caused by multiple bacterial agents, as they comprise phages with various host ranges. Current advancements in this area of study include the development of phage cocktails [11], phage engineering [12] and combination therapies [13].

As the study of phage therapy advances while MDR bacteria continue their rapid emergence, it is important to address the gaps in knowledge of phage host ranges and how these affect the effectiveness of phages against MDR bacteria. This review covers an overview of bacteriophages, the importance of bacteriophages against MDR bacteria, the interactions between phages and their hosts, the diversity of the phage host range, and the future development of phages in terms of host range expansion.

## 2. Survey Methodology

To provide a comprehensive literature review on how phage host range affects phage therapy against MDR infections, a range of information and data were collected using search engines such as “PubMed (Bethesda, MD, USA)”, “Scopus (Amsterdam, The Netherlands)”, “Ovid MEDLINE (New York, NY, USA)” and “Google Scholar (Mountain View, CA, USA)”. Journal articles specific to the keywords multidrug-resistant (MDR) bacteria, bacteriophage/phage therapy, bacteriophage/phage host range, narrow host range, broad host range, polyvalent and monovalent were analyzed during the write-up of this review. A wide variety of relevant articles were sought from the time of general phage discovery (1915) to the current advancement in phages (2023). These articles were then categorized through Mendeley software (Amsterdam, The Netherlands, https://www.mendeley.com/?interaction_required=true) and gradually organized in order to make sure that our review was thorough, rational and balanced. Finally, the article was written based on the compiled findings.

## 3. Brief Overview of Bacteriophages and Their Interactions with Bacterial Hosts 

Bacteriophages (or phages) are viruses that specifically infect and kill bacteria at the end of the phage infection cycle, causing bacterial cell death and lysis. In early 1896, a British bacteriologist, Ernest Hankin, noticed the presence of antibacterial activity in the Ganges and Jumna rivers (India). An unknown substance that was found to be heat-labile and could pass through very fine porcelain filters appeared to exhibit antibacterial properties, reducing the spread of *cholera* infection in villages [14]. Two years later, Nikolay Gamaleya, a Russian bacteriologist, noticed a similar event while studying *Bacillus anthracis* [15]. However, the “official” discovery of bacteriophages occurred in 1915 and 1917, when British pathologist Frederick Twort described a similar phenomenon while studying *staphylococci* and suggested that viruses might be responsible for this antibacterial activity [16], and French microbiologist Felix d’Herelle published a paper clearly defining the viral nature of the invisible anti-Shiga microbe and named it a “bacteriophage”—a combination of the words “bacteria” and “phagein” (which means to eat or devour in Greek) [17].

Like other viruses, bacteriophages are said to be obligate intracellular parasites. Despite having the genetic material to direct replication, they have to rely on the reproductive machinery of the bacterial host. Generally, each phage consists of a single type of nucleic acid in the head, either double- or single-stranded deoxyribonucleic acid (DNA) or ribonucleic acid (RNA), which is circular or linear [18]. The size of the genome that determines all the properties of the phage can range from a few to hundreds of kilobases, and they are enclosed within a protein coat—a capsid [19]. 

In 1967, Bradley classified phages into six basic types (Groups A to F) based on their morphological characteristics and nucleic acid composition: filamentous phages, icosahedral phages, and tailed phages, with ssRNA or ssDNA [20]. According to Bradley, Group A phages have long tails with a contractile sheath; Group B and C phages have long and short non-contractile tails, respectively; Group D and E phages lack a tail, but the former have big capsomeres and the latter have small capsomeres; and Group F phages are filamentous, with flexible filaments. This classification scheme still serves as the basis for the current taxonomy of phages. In 1995, the International Committee on the Taxonomy of Viruses (ICTV) classified tailed phages into three families: Myoviridae, Siphoviridae and Podoviridae, corresponding to Groups A, B and C of Bradley’s classification [21]. Phages were then classified by the ICTV into one major order, 19 families, and 31 genera [22]. It is worth mentioning that most of the phages (96%) identified were tailed dsDNA phages belonging to the families Myoviridae, Podoviridae or Siphoviridae of the same order Caudovirales, while only 3.6% of such phages were polyhedral, filamentous, or pleomorphic [23]. 

### 3.1. Phage Life Cycle and Its Relations to Phage Host Range

**Figure 1:**
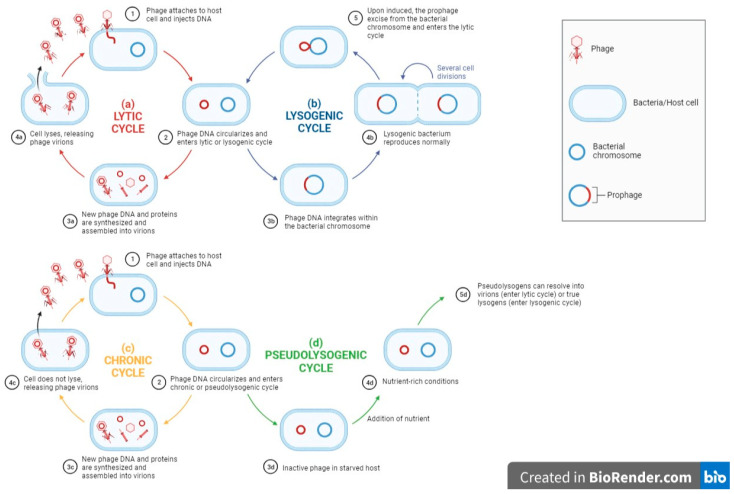
A flow diagram showing general phage–host interactions (**a**) lytic cycle, (**b**) chronic cycle, (**c**) lysogenic cycle, (**d**) pseudolysogenic cycle. Adapted from “Lytic and Lysogenic Cycle”, by BioRender.com (2021). Retrieved from https://app.biorender.com/biorender-templates (accessed on 23 May 2023).

Phage host ranges are determined by their ability to replicate in and kill their bacterial host. Therefore, it is vital to study all aspects of the phage-host interactions, not just the attachment, so as to achieve a greater understanding of the issues of broad and narrow host ranges. After the phage binds to the host bacteria, it will initiate different phage–host interactions, namely, the lytic cycle, lysogenic cycle, pseudolysogenic cycle and chronic cycle [24]. Virulent phages usually take control of the host metabolism after infection and use it for replication and synthesizing new phage particles. The release of viral progeny from the host cell by lysis will result in a new lytic cycle carried out by the viral progeny. On the other hand, the lysogenic cycle involves the integration of the viral nucleic acid into the bacterial genome, forming a prophage. Together with the bacterial genome, these prophages are transmitted to the host descendants until the lytic cycle is induced. A pseudolysogenic cycle is a variant of the lysogenic cycle where the phage is inactivated within the host. This usually occurs during starvation. Finally, in the chronic cycle, phages replicate in the host and exit the host cell by budding instead of by lysis, thus sparing the host and resulting in continuous phage production. The above phage–host interaction is briefly summarized in Figure 1.

The attachment stage in the cycles summarized above is often the focus in studies of the molecular mechanisms of broad-host range phages (see below). However, following adsorption, the phage must go on to replicate successfully in the host, which may depend upon the phages’ genetics and regulatory mechanisms. In addition, host range modulation may also largely depend on the different proteins present, as well as the host receptor-binding proteins or RBPs adaptations. 

### 3.2. Bacteriophage Adsorption and Receptors Present in MDR Bacteria

Commonly, the first stage of both lytic and temperate infections begins with adsorption. This involves the specific phage–host interaction between the RBPs or adhesins of a phage and receptors on the bacterial cell surface [25]. Tailed phages use their tail adhesins (including base plate proteins and tail fibers) to interact with bacterial receptors and thus penetrate the bacteria cell surface to eject phage DNA, whereas tail-less phages use capsid proteins such as spike protein G, capsid protein F, and DNA pilot protein H to carry out the tail-associated function to enable subsequent DNA ejection [26]. In tailed phages, there are three steps involved in phage adsorption: initial contact with bacterial surface receptors, followed by reversible binding, and finally irreversible binding, during which the phages can no longer dissociate from the bacterial cell surface [27,28]. The host specificity of the phages is determined at this stage because the subsequent steps in the lytic cycle can only proceed if the phage successfully attaches to and penetrates the bacterial cell; the identification of cell surface receptors is therefore crucial. There are various cell surface receptors on Gram-positive bacteria and Gram-negative bacteria that can be targeted by the phage during adsorption. These are either constituents of bacterial cell walls such as proteins, polysaccharides, lipopolysaccharides, and carbohydrates, or protruding structures such as pili, flagella, and capsules [29]. 

In Gram-positive bacteria, the common surface receptors that can be recognized by the phage are on the one hand peptidoglycan or murein, and on the other teichoic acid, both important components of the bacterial cell wall [28]. In MDR Gram-positive strains such as MRSA, meanwhile, studies have reported that the wall teichoic acid acts as the receptor for broad-host range phage ΦMR003 to recognize and bind [30]. Another phage, ΦSA039, that can infect MRSA also recognizes wall teichoic acid as the receptor, though with the difference that ΦSA039 also requires the β-GlcNAc residue on wall teichoic acid for phage adsorption [31]. In addition, the membrane proteins PIPEF (phage infection protein of *Enterococcus faecalis*) and *enterococcal* polysaccharide antigen (Epa) have been identified as receptors of Vancomycin-resistant *enterococci* (VRE) and can be attached by a collection of *enterococcus*-specific phages [32]. 

In Gram-negative bacteria, the typical surface receptors that can be attached by the phage are lipopolysaccharides and proteins located on the outer membrane, flagella, pili and capsules [28]. In the MDR Gram-negative *Shigella* species, it was recently discovered that a phage called Sfin-1 can infect *Shigella flexneri*, *Shigella sonnei* and *Shigella dysenteriae* by recognizing a lipopolysaccharide O-antigen for adsorption [33]. Moreover, capsular polysaccharides consisting of tightly packed repeating K units have been identified as the receptors of carbapenem-resistant *A. baumanni* for the *A. baumannii*-specific phage øCO01 to adsorb [34]. Furthermore, in a personalized nebulized phage therapy study, two *Pseudomonas* phages LPS-5 and TIVP-H6 were shown to bind to lipopolysaccharides and type-IV pili, respectively, of MDR *P. aeruginosa* [35]. Finally, the phages Pharr and ΦKpNIH-2 have been shown to infect MDR *K. pneumoniae* by binding to their capsular polysaccharides and outer membrane porin OmpC or lipopolysaccharides, respectively [36].

In brief, the potential of a phage to affect a bacterial population and the susceptibility of a bacterium to phage infection largely depends on the host range of the phage, which in turn depends primarily on its adsorption properties [37]. Both bacterial and phage characteristics, such as the nature and location of bacterial cell surface receptors and RBPs, can limit the host range of phages, making them specific to one bacterial species or even to only a few strains within a bacterial species. Before starting phage therapy, phage typing or applying phages in the food industry, it is therefore crucial to identify their specificity range against the pathogenic bacteria in question. 

### 3.3. Phage–Host Interaction 

As mentioned earlier, phages can kill antibiotic-resistant bacteria in the final stage of the infection cycle. The life cycle of phages determines the role they play and how they can be applied in different approaches. There are two main modes of infection by bacteriophages—lytic (or virulent) infection and temperate infection, carried out by lytic and temperate phages, respectively. In lytic infection, phages infect and kill their bacterial host cell in four stages: adsorption on host cell surface, insertion of phage DNA, replication of phage DNA, lysis of host cell, and releasing of newly formed phages into the environment [38]. In contrast, there are two possible outcomes in a temperate infection: either the host cell lyses and releases newly formed phages, similar to a lytic infection, or the phage DNA may integrate into the bacterial chromosome to become a prophage, a process known as lysogeny [39]. This prophage is non-infectious until it is induced by UV irradiation or chemicals, inducing it to enter a lytic cycle [40]. The key thing to note here is that phage induction is primarily caused by DNA damage. Another potential outcome of lysogeny is lysogenic conversion, i.e., the alteration of bacterial phenotypes and behaviors with virulence or other determinants such as toxin genes (usually acquired from temperate phages). These latter may protect bacteria from phage reinfection [41]. Therefore, lytic phages are usually the first choice for phage therapy, while the use of temperate phages is much less common.

However, Edgar et al. (2012) attempted to use phage-based delivery systems, where temperate phages were used as vehicles to deliver DNA encoding drug-sensitizing genes to bacteria, so as to increase bacterial susceptibility to antibiotics [42]. They were successful in doing this: streptomycin- and nalidixic acid (NA)-resistant *E. coli* K-12 lysogenized with designed phages carrying the genes rpsL and gyrA (confer sensitivity to streptomycin and NA, respectively) showed restored antibiotic sensitivity. Additionally, Park et al. (2017) integrated the CRISPR/Cas9 system into the genome of a temperate phage (φ SaBov) in the hope of improving the current limitations of phage-based delivery systems [43]. Besides managing to expand the host specificity by complementing the tail fiber protein of the phage, they also managed to remove the virulence factor genes from the *S. aureus* strain (RF122) to prevent contamination and the spread of virulence genes by transduction. The 10 superantigens (e.g., sec, seg and selo) and 11 cytolysins (e.g., hla, hlgA and lukD) of RF122 were removed from the chromosome of *S. aureus* using allelic exchange by a modified shuttle vector system (pMAD-secY system). The results show that the phage lysates generated from this modified strain (R122-19Δnuc) did not cause any cytotoxicity or superantigenicity [43].

These experiments suggest that genetically engineered temperate phages may be used as gene delivery vehicles to create programmable gene-specific antimicrobials that are less likely to drive resistance than antibiotics. However, not much is known about the potential limitations of this study—for example, whether such alterations of bacterial genes might alter microbiological niches in the environment. More studies are thus required to fill the gaps in this research area, so that temperate phages can remain an option for phage therapy to reduce our reliance on lytic phages.

## 4. Broad-Host Range and Narrow-Host Range Phages

Phages can be broadly categorized into either monovalent or polyvalent phages depending on their respective host range spectrums. Monovalent phages are defined as phages that have a narrow host range and are specific to a single bacterial genus, whereas polyvalent phages are phages with a broad host range that are specific to more than two genera [10]. Most reported phages are species-specific or strain-specific, and thus are said to exhibit only narrow specificity.

However, the terms “broad host range” and “narrow host range” are often overused and misused within the scientific community, thereby reducing their usefulness in defining host ranges. Yu et al. (2016) [44], Duc et al. (2020) [45] and Sui et al. (2021) [46] described broad host ranges as phages that can infect multiple bacterial species. However, the same term is also sometimes used to describe phages that infect various strains of the same bacterial species [47,48,49]. Furthermore, although the term “polyvalent” was earlier used to describe phages active on different bacterial genera by Ackermann and Dubow (1987), it has often been used similarly to broad-host range phages more recently [50]. Some examples where the authors have described broad-host range phages as polyvalent include Hamdi et al. (2017) [51] and Kim, Adeyami, and Park (2021) [10].

The terms “narrow” and “broad” host range are often used interchangeably, but sometimes inconsistently, with the terms “monovalent” and “polyvalent” as well. Apart from variations in the terminologies used, there is also currently no proper standard as to the amount or percentage (%) of strains/species tested that must be infected for a phage to be deemed “broad-host range”. For instance, Gupta and Prasad (2010) described their P-27/HP phage as polyvalent when it was found to infect 60% of the 28 *S. aureus* isolates [52]. Kim, Adeyami and Park (2021), on the other hand, also described their phage (KFS-EC3) as having a broad host range when it was shown to infect only 7 out of 57 bacterial strains: 3 strains of *E. coli* (*E. coli* O157:H7, *E. coli* O157:H7 ATCC 10536, and *E. coli* O157:H7 204p), 3 strains of *Salmonella* spp. (*S. Enteritidis*, *Salmonella Mission* and *Salmonella Senftenberg*), and finally *S. sonnei* ATCC 9290 [10]. Moreover, researchers often develop their own bacteria collection into a standard according to previously described literature and newly isolated strains or species, because there are no general standards in the numbers of bacteria species or strains tested and bacterial species collections. All this leads to inconsistency in phage host range descriptions.

### Advantages and Limitations of the Different Phage Host Ranges in the Treatment of MDR Bacteria

Most of the phages reported previously are species-specific or strain-specific, exhibiting only narrow specificity. Indeed, narrow-host range phages that only infect a single species were favored in the past, as a means of preventing the phage virus from disrupting the human microbiome [53]. Such narrow-host range phages were isolated by Auad et al. (1997), Lu et al. (2003) and Lin et al. (2011) using a single bacterial host [54,55,56]. However, using this type of phage greatly limits the application of phage therapy, since many infections are caused by multiple bacteria. For instance, perirectal diseases are often caused by multiple bacteria such as *E. coli*, *S. aureus*, and *Streptococcus* species [57]. To complicate the issue further, various infections such as sepsis and abscesses caused by hepatic artery catheterization and biliary drainage are often linked to multiple MDR bacteria. For example, liver abscesses are usually caused by a mixture of bacteria including *E. coli*, *K. pneumoniae*, and *Enterococci*. Recently, both MDR and hypervirulent *K. pneumoniae,* as well as *E. coli,* were found to cause these infections at the same time [58,59].

In addition, phages with a narrow host range might also be ineffective against MDR bacteria on their own. They are therefore often combined with other narrow-range phages to develop a phage cocktail. Law et al. (2019) recently attempted phage therapy in cystic fibrosis on a patient with severe respiratory failure [60]. The patient was infected with two strains of MDR *P. aeruginosa,* and as a result required mechanical ventilation. A cocktail containing four presumably narrow-host range phages was used as treatment, along with other antibiotics, and the patient was successfully cleared of *P. aeruginosa* after eight weeks. In conclusion, phages with a narrow host range are often limited to only one species of bacteria, and thus are not ideal for use on their own in phage therapy.

Broad-host range phages, on the other hand, are considered more useful in phage therapy as they can kill multiple species of bacteria, making them similar to broad-spectrum antibiotics. These phages may thus be able to treat bacterial infections caused by the same species but different strains empirically, thereby avoiding the need to test the infecting strain for susceptibility. vB_EcoM_LNA1 (A1) [61], JD419 [62] and PAK-P3 and P3-CHA [63] are among the phages that can infect multiple strains.

Broad spectrum can also mean that phages can target bacteria across species. For instance, Acpi12 [64] and ΦSER1 [65] are phages that have shown that they can target multiple bacterial species, such as *Klebsiella* spp. and *E. coli*. Such phages were previously rare, but have been slowly emerging in greater number in recent years (this will be discussed in detail in the later sections), probably as a result of the increased use of multiple host receptors to isolate them. Phages have generally been isolated via a single host strain and, although this approach can sometimes yield a broad-host range phage, most such phages turn out to be “narrow-ranged”. For instance, 16 different host strains were used to isolate and broaden the host range for *P. aeruginosa* by spot assay testing [12].

Another advantage of phages with broader host ranges is that they reduce problems of mismatched host and phage combinations. This in turn minimizes the risks of failure in disease treatment [53].

In sum, a phage’s host range is a vital factor in determining how, and how effectively, it can be used for phage therapy. Thus, it is important to consider and research further into the advantages and limitations of both narrow and broad-host range phages in order to develop suitable treatments for MDR bacterial infections, as well as other diseases.

## 5. Importance of Phage Applications in the Following

### 5.1. Clinical MDR Bacterial Treatment

Shortly after d’Herelle discovered bacteriophages while studying patients recovering from bacillary dysentery in 1917, he conducted phage therapy on a 12-year-old severe dysentery patient, and the treatment was very successful [66]. This attempt provided a new insight for other scientists into the potential therapeutic applications of phages. Since then, similar studies have also been performed with other bacterial infections, such as typhoid fever, *staphylococcus* infections and urinary tract infections caused by colon bacteria. The outcomes of these have been generally positive, despite a few inconsistent results occasioned by a lack of understanding of the biological nature of phages [67].

In the early 1940s, the first antibiotic, penicillin, was developed and used as a medical treatment [68]. As antibiotics possess a broad spectrum of activity, they are very effective in controlling bacterial infections. As a result, the usage of antibiotics in bacterial infection treatments gradually became the gold standard around the world, leaving phage therapy research far behind. In later decades, however, multiple bacterial infections have become progressively more difficult to treat due to the emergence of MDR bacteria, such as *A. baumannii* [69], methicillin-resistant *Staphylococcus aureus* (MRSA) [70], and vancomycin-resistant *enterococcus* (VRE) [71], leading to higher morbidity and mortality rates. The current crisis has prompted researchers to look for alternatives, including revisiting phage therapy as a “new” and potentially effective treatment to tackle different bacterial infections in both animals and humans [72].

Phages have an antibacterial mechanism that differs from that of antibiotics, making them potentially more effective against MDR bacterial strains [73]. Phage therapy also offers various other advantages over antibiotics. One is that, as species-specific and self-amplifying drugs, the taking of phages does not cause toxic effects or immunological complications, a crucial factor for people with impaired immune systems [37,74,75,76]. Recent clinical trials using phage therapy have shown that a variety of therapeutic bacteriophages can successfully treat burns [77], mycobacterial infection [78], venous leg ulcers [79], chronic otitis [80], urinary tract infection [81] and diarrhea [82]. In the specific case of MDR bacteria, a lytic bacteriophage called Abp9 was proven to be effective against the MDR *A. baumannii* strain AB_ZY_9, and could therefore be used to treat patients with *A. baumannii* infections [83]. In addition, there is also a *staphylococcal* bacteriophage called Sb-1, which can infect MDR *Staphylococcus aureus* and so be used in the treatment of diabetic foot ulcers (DFUs) [84]. Intriguingly, an MDR *A. baumannii* bacteriophage called p54 was shown to have significant bactericidal activity not only against MDR *A. baumannii* itself, but also against other Gram-negative bacteria such as *K. pneumoniae*, *P. aeruginosa*, and *Escherichia coli* [85]. Another *A. baumannii* bacteriophage, PD6A3, can also inhibit other bacteria such as *E. coli*, *Enterococcus faecium* and *P. aeruginosa* [86]. In another development, Pallavali et al. (2021) discovered four lytic bacteriophages, namely, vB_PAnP_PADP4, vB_KPnM_KPDP1, vB_SAnS_SADP1, and vB_ECnM_ECDP3, that can effectively tackle MDR *P. aeruginosa*, *K. pneumoniae*, *S. aureus* and *E. coli,* respectively, indicating their potential to be used in therapy as an alternative approach to antibiotics [87]. All that said, despite these various successful ventures, phage therapy still needs to proceed with caution.

Finally, another potential use of phages is phage typing. In this process, phages, being species-specific, are used as phenotyping tools to identify and differentiate a single strain of bacteria. Until recently, the use of phages as phenotyping tools in MDR bacteria was not well developed. In early 2005, for example, studies reported that only 4 out of 23 lytic phages used for phage typing were active against MDR *staphylococcus* strains, suggesting that MDR bacteria are much less typable than strains that are susceptible to phages [88]. However, a study has demonstrated that phage typing can be helpful in the epidemiological surveillance and outbreak investigation of MDR *Salmonella Typhimurium* [89]. In addition to *S. Typhimurium*, a set of 23 phages was also used in the phage typing of MRSA for epidemiological monitoring, with a majority of the MRSA strains (84%) proving to be typable [90]. Furthermore, phage typing in conjunction with antibiotic susceptibility testing has also been shown to facilitate in the identifying of different strains of MDR *K. pneumoniae* that co-occur at an infection site [91].

### 5.2. Combating MDR Bacteria in the Agriculture and Food Sectors

The application of phages in other sectors such as the food, livestock and farming industries has also shown promise. One of the benefits of phages is that they do not alter the properties of food products, such as the taste, color, smell, or texture [92]. This allows phages to be used in the food industry as biological controls to eliminate bacterial contaminants that can lead to food spoilage, as well as the production of biofilms that can get onto food products and reach customers, and the possibility of microbial foodborne infections [93]. Recently, many novel bacteriophages have also been discovered and identified as biocontrol agents of MDR bacteria. For instance, a polyvalent bacteriophage within the family Siphoviridae, termed phiLLS, has been shown to inhibit the growth of MDR *E. coli* and has a wide range of activity, making it a potential biocontrol agent [94]. Intriguingly, Tian et al. (2022) developed a biomolecular-friendly high-throughput preparation method to synthesize M13, a detachable phage microgel. This microgel can prevent the growth of MDR *E. coli* O157:H7 in lettuces and meats, hence demonstrating its excellent antimicrobial activity [95]. Prior to this, studies conducted by Le et al. (2018) showed that a phage cocktail consisting of five *E. coli*-specific phages and one *Salmonella* phage can effectively control the growth of MDR enteric bacteria such as *E. coli* and *Salmonella enterica* on edible oysters [96], suggesting that this phage cocktail could be used to prevent MDR pathogens from contaminating seafood during processing.

To sum up, bacteriophages can be used as therapeutic agents against MDR bacteria in the healthcare sector, as phenotyping tools in the investigation of MDR bacteria outbreaks, and as biocontrol agents in food processing as shown in Table 1. All these phage applications are based on the binding of phages to receptors on the surface of specific bacteria. This highlights the importance of improving our understanding of the interactions between hosts and phages, to allow us to develop further successful phage applications in the fields of health, ecology, and food.

## 6. Current Studies of Broad-Host Range Phages against MDR Bacteria

MDR bacterial infections can have serious implications for human health, and thus pose a major challenge for clinical and pharmaceutical research. Phage therapy has already been proven to be an effective and promising alternative treatment for MDR bacterial infections. For example, a broad-host range lytic phage named SHWT1 has been found to be effective against MDR *Salmonella* including *S. Enteritidis* and *S. Typhimurium*. Studies have also been conducted wherein SHWT1 showed successful protection against mice infected with the two previously mentioned *Salmonella* spp. [47]. Other examples include ΦSER1, which was found to lyse different species of bacteria including *Klebsiella* spp., *E. coli*, *Pseudomonas* spp., *Enterobacter* spp., *Serratia* spp., *Citrobacter* spp. and MDR *Pseudomonas* [65]. The findings of this research are particularly significant since they suggest that phages might be used against Metallo-β-lactamases (MBL) and Extended Spectrum Beta-Lactamase (ESBL) strains, which can cause bacterial infections such as sepsis and urinary tract infections (UTI).

In addition, phages have also been found to be potentially effective in the treatment of cystic fibrosis. Morello et al. (2011) tested PAK-P3 and P3-CHA phages on MDR *P. aeruginosa* cystic fibrosis strains using a mouse lung infection model [63], and found that these two phages have high effectiveness in the treatment of lung infections when administered intranasally.

A case study was also conducted with six phages (Kp152, Kp154, Kp155, Kp164, Kp6377, and HD001) being used to develop a cocktail against extensively drug-resistant *K. pneumonia* (ERKp) in urinary tract infections [97]. Cocktails I and II used in this study led to the emergence of phage-resistant mutants in the patients. The subsequently produced Cocktail III, on the other hand, combined with non-active antibiotics proved to be an effective treatment, able to lyse all three strains of ERKp isolated from the patient-CX7224, CX8070 and CX10301. The study reported that the patient’s pathogenic ERKp were eliminated with no signs of recurrent UTI symptoms. Nor were there any signs of recurrence after 6 months post-treatment. This last study particularly highlights the positive combined effect that can be achieved by using several phages as a cocktail in combating MDR bacteria. Such cocktails have by their nature a broad host range specificity, making them more effective against target bacteria. A compilation of polyvalent phages capable of infecting various species of MDR bacteria is shown in Table 2.

## 7. Future Directions

One of the major limitations of phage therapy against MDR bacteria is that phages that have been isolated often have a narrow host range and serotype specificity, which can reduce the effectiveness and coverage of the phage in treatment [103]. To overcome the limitations of such individually narrow-host range phages, phage cocktails have been increasingly developed that consist of several phages with different receptors and features. These cocktails contain various types of phages that can complement each other and help target the same bacteria species and strains, hence slowing down the emergence of phage-resistant bacteria [104,105]. One such cocktail was recently developed by Martins et al. (2022) [11]. They managed to combine eight previously identified phages into a cocktail named Katrice-16, which was then tested and proved to be effective against MDR *K. pneumoniae*. Other examples of phage cocktail development against MDR bacterial diseases can be found in research conducted by Pereira et al. (2016), Shahin et al. (2020), and Haines et al. (2021) [105,106,107]. In sum, the development of phage cocktails combining multiple narrow-host range phages can to some extent overcome the shortcomings of narrow-host range phages.

The narrow range of many phages renders them less effective in treating not only chronic diseases caused by MDR bacteria, but also diseases caused by other bacteria. Another way to address these limitations is to modify phages themselves through phage engineering. This has recently been achieved to produce phages with more desirable characteristics, such as having a broader host range or better lytic activities [103]. The production of toxin proteins, the alteration of host recognition receptors, and the disruption of bacterial phage resistance pathways are a few examples of how genetic engineering can be applied.

One way to achieve host range expansion is to modify the phage’s tail fiber protein. Successful phage infection involves a phage binding to the bacterial surface, which relies heavily on cross-linking between phage-binding proteins on the tail fiber and host receptor proteins. The host range of most wild-type phages tends to be limited to specific bacteria due to the size, shape, and location of their phage fiber binding proteins [12]. An early example of tail fiber protein modification was carried out by Lin et al. (2012), who replaced a partial T3 phage tail fiber gene with one from a T7 phage [108]. The recombinant phage thus created a broader host range and better adsorption efficiency than the T3 and T7 wild types on their own. However, frequent mutations in receptor proteins allow infected bacteria to survive even after modified phages have expanded their host range. Through a high-throughput sequencing study of the T3 phage mutant in a coculture system using the T3 phage and BL21, Yehl et al. (2019) found that most phage mutations occur in the host range-determining regions (HRDR) [109]. They then created a mutant library in these regions, which showed that such mutants can reduce the host bacteria number at least five times more effectively than the wild-type T3. The mutants in the library also inhibited bacterial growth in vitro for approximately a week, with no evidence of phage resistance.

Another developing area of work is the use of chemical engineering to modify phages. This has been used for example to crosslink phage coats with antibiotics, antimicrobial peptides, heavy metal ions, and photothermal materials. Contemporary research on phage chemical modification has mainly focused on increasing bactericide activity. Nanoparticles have become an increasingly popular biological agent to try to combat the current spread of MDR bacteria. For instance, a filamentous phage M13 was induced to adsorb silver nanoparticles on its coat protein via ionic binding, and this modified phage showed that it could kill *Fusobacterium nucleatum* in colorectal cancer tumor tissues accurately [110]. Other chemical modifications include using AIEgens, pheophorbide a (PPA) and indium tin oxide (ITO), as reported by He et al. (2020), Dong et al. (2018) and Anany et al. (2011), respectively [111,112,113].

Drawing on the above, another area of growing interest is phage training—a novel method for producing efficient phages that takes advantage of wild-type phages’ evolutionary reaction to bacterial resistance. This area can be divided into natural, enforced and engineered. Natural training is where both phage and bacteria develop without bias and exist in nature without human intervention. Enforced training on the other hand is where the selection is deliberately biased by treating the phage with other agents, such as silver nanoparticles or antibiotics [114,115].

Two further areas of advances in recent years include the use of combination therapies involving both phages and antibiotics and delivery systems. On the first of these, a study showed that a combination of an antibiotic (ampicillin) and a phage cocktail (*Shigella*-specific bacteriophages), named ShigActive^TM^, was able to effectively reduce the amount of *Shigella* in mice [116]. On delivery systems for phages, a recent breakthrough was the use of a liposome as a delivery vehicle to allow the phage to act inside the bacterial cell and avoid being targeted by anti-phage antibodies [13].

Looking ahead, human models would also benefit from further development to improve our ability to determine the efficacy of phages through research or clinical trials. One advance in this area was the development of a new human intestinal organoid-derived infection model to study the efficacy of a novel phage against *S. flexneri*. This new model enabled researchers to demonstrate that a novel phage could prevent phage-specific strains and other isolates of the same species from adhering to and invading epithelial cells [117].

As the developments described above show, many of the disadvantages of phage therapy can now be overcome with the advancement of technology. Moreover, more and more researchers have steadily shown positive results in the application of phages in MDR treatment. The use of phages as a preventive measure and the development of vaccines based on phages or phage products adds a new dimension to the fight against MDR bacteria. Beyond this, the active involvement of more patients with MDR bacterial diseases and preferably a large-scale trial of phage therapy against MDR bacteria could help to improve the acceptance of phage therapy as a common treatment. The scientific community and government should also seek to stimulate international collaboration among national phage banks, libraries, and directories so as to improve the current knowledge of phage therapy [6]. Finally, to further extend and exploit this potentially fruitful area of knowledge, it is vital to raise public awareness of phage therapy, as well as expand the availability of phages and phage therapy centers.

## 8. Conclusions

Despite the many and growing advantages of phage therapy, there is still a long way to go before phages can be considered a “magic bullet” in treating infections. A number of parameters not yet accurately analyzed or determined in clinical trials, such as pharmacological aspects, the frequency and duration of treatment, optimal dosage, and the pathway of administration, stand in the way of this. Other potential challenges include the complication of pharmacokinetics and pharmacodynamic phage treatments, patenting, manufacturing, and administration.

In all this, the host range of phages undeniably plays a critical role in determining the effectiveness of phage therapy against MDR bacterial diseases. As this study has shown, it is therefore essential to expand the knowledge of current and novel phages, and in particular to investigate how to expand further the host range of certain potential phages in order to extend and exploit this potentially fruitful discovery.

## Figures and Tables

**Table 1:** Summary of recently discovered bacteriophages that are effective against MDR bacteria serving as therapeutic agents, phenotyping tools or biocontrol agents.

No.	Phage(s)/Phage Cocktail Name	Effective Against	Application	Reference
1.	Abp9	MDR *A. baumannii* strain AB_ZY_9	Therapeutic agent	[83]
2.	Sb-1	MDR *S. aureus*	Therapeutic agent	[84]
3.	p54	MDR *A. baumannii*, *P. aeruginosa*, *K. pneumoniae*, and *E. coli*	Therapeutic agent	[85]
4.	PD6A3	MDR *A. baumannii*, *E. coli*, *E. faecium* and *P. aeruginosa*	Therapeutic agent	[86]
5.	DT104 phage types	MDR *S. Typhimurium*	Phenotyping tool	[89]
6.	5 phage groups consisting of 23 phages	Methicillin Resistant *S. aureus*	Phenotyping tool	[90]
7.	JIPh_Kp 192– 199, and JIPh_Kp 202	MDR *K. pneumoniae*	Phenotyping tool	[91]
8.	phiLLS	MDR *E. coli*	Biocontrol agent	[94]
9.	M13	MDR *E. coli* O157:H7	Biocontrol agent	[95]
10.	Phage cocktail consisting of ΦEco1, ΦEco2, ΦEco3, ΦEco5, ΦEco6 and ΦS1	MDR enteric bacteria such as *E. coli* and *S. enterica*	Biocontrol agent	[96]

**Table 2:** A compilation of recent studies of phages with broad host range. According to the authors, these include both effective against different strains of the same species [A] and effective against different species of bacteria [B] in the treatment of the MDR bacterial diseases listed in the table.

No.	Phage/s	Host Strain	Against	Effectiveness	Description/ Remarks	Reference
**In vitro**						
1.	vB_EcoM_LNA1 (A1)	*E. coli* K12 MG1655 w/RP4 plasmid	*E. coli* K12 MG1655 w/RP4 plasmid, *E. coli* K12 MG1655 w/pMS6198A plasmid, uropathogenic *E. coli* (UPEC) S79EC, UPEC S129EC	Phage exhibited broad host range recognition and strong infectivity against UPEC strains as demonstrated by a large burst size and extended bacterial growth suppression.	[A]	[61]
2.	SHWT1	*Salmonella pullorum*	MDR *Salmonella* (*S. Pullorum*, *Salmonella Gallinarum*, *S. Enteritidis*, *S. Typhimurium*, *Salmonella Derby*, *Salmonella London*, *Salmonella Typhi*, *Salmonella Heidelberg*, *Salmonella Paratyphi B*)	Phage had a short latent period (5 min) and an average burst size of 146.6 ± 10.8 PFUs/cell. It retained lytic activity for at least 60 min at temperatures ranging between 4 and 65 °C and remained stable at pH 3 to 12.	[A]	[47]
3.	JD419	*S. aureus*	MDR clinical *S. aureus* strains	A temperate phage that is stable at pH 6 to 8 and below 50 °C. Rapid replication and lysis of host strains were observed. No virulence or antibiotic resistance genes.	[A]	[62]
4.	AP025 and AP006	*P. aeruginosa* PAO1/ *P. aeruginosa* ATCC9027 /clinical isolate	MDR *P. aeruginosa*	AP025 and AP006 phages exhibited a good infectivity rate (host range infectivity) of 39% and 30%, respectively, against MDR strains.	[A]	[98]
5.	AP22	*A. baumannii*	Genotype-varying MDR clinical *A. baumannii* strains	Phage exhibits rapid adsorption (>99% adsorbed in 5 min), a large burst size (240 PFU per cell), and stability in a wide range of pH. Infect and lyse 68% of MDR *A. baumannii.*	[A]	[99]
6.	C11S1A	*E. coli*	MDR *E. coli* in East Africa	Phage killed all 23 *E. coli* strains. Highly efficacious at 37 °C and pH 7.4.	[A]	[100]
7.	ΦSER1	*Serratia*	*E. coli*, *Enterobacter* spp., *Klebsiella* spp., *Serratia* spp., *Pseudomonas* spp., *Citrobacter* spp., MDR *Pseudomonas*	85% effectiveness in terms of host range when compared with other phages.	[B]	[65]
**In vivo**						
8.	SHWT1	*S. pullorum*	MDR *S. enteritidis and S. typhimurium*	Reduced mice mortality when phage treatment was introduced. Survival rate of *S. Enteritidis* infection: 40% Survival rate of *S. Typhimurium* infection: 80%.	[A]	[47]
9.	AP025 and AP006	*P. aeruginosa* PAO1/*P. aeruginosa* ATCC9027 /clinical isolate	MDR *P. aeruginosa*	A single dose of phages at higher concentrations, bacteria:phages at 1:10 and 1:100 were effective in eliminating bloodstream infection and achieving 100% mice survival.	[A]	[101]
10.	PAK-P3 and P3-CHA	*P. aeruginosa*	MDR *P. aeruginosa* cystic fibrosis strains	A curative treatment (one single dose) administered 2 h after the onset of the infection allowed over 95% survival. A four-day preventive treatment (one single dose) resulted in 100% survival.	[A]	[63]
11.	øKp_Pokalde_002	*K. pneumoniae*	Carbapenem-resistant *K. pneumoniae* (Kp56)	Bacterial count significantly decreased in blood and other organs after 24 h of phage administration. Phage exhibited rapid clearance and did not stimulate proinflammatory cytokines. There is also a significant reduction in proinflammatory cytokines caused by bacterial infection, reducing tissue inflammation.	[A]	[102]
**Case reports**						
12.	Cocktail III (Kp152, Kp154, Kp155, Kp164, Kp6377, and HD001)	*K. pneumonia*	Extensively drug-resistant *K. pneumonia* (ERKp) in UTI	Phage-resistant mutants emerged when Cocktails I and II were used. After phage therapy (Cocktail III) combined with non-active antibiotics treatment, the patient’s pathogenic ERKp was completely eliminated and there are no recurrent UTI symptoms. No signs of recurrence for 6 months of follow-up.	[A]	[97]

## Data Availability

Not applicable.

## References

[1] Tommasi R., Brown D.G., Walkup G.K., Manchester J.I., Miller A.A. (2015). ESKAPEing the labyrinth of antibacterial discovery. Nat. Rev. Drug Discov..

[2] Reygaert W.C. (2018). An overview of the antimicrobial resistance mechanisms of bacteria. AIMS Microbiol..

[3] Tanwar J., Das S., Fatima Z., Hameed S. (2014). Multidrug Resistance: An Emerging Crisis. Interdiscip. Perspect. Infect. Dis..

[4] World Health Organization (2021). Antimicrobial Resistance. https://www.who.int/news-room/fact-sheets/detail/antimicrobial-resistance#:~:text=The%20main%20drivers%20of%20antimicrobial,access%20to%20quality%2C%20affordable%20medicines%2C.

[5] Paul V.D., Sundarrajan S., Rajagopalan S.S., Hariharan S., Kempashanaiah N., Padmanabhan S., Sriram B., Ramachandran J. (2011). Lysis-deficient phages as novel therapeutic agents for controlling bacterial infection. BMC Microbiol..

[6] Nagel T., Musila L., Muthoni M., Nikolich M., Nakavuma J.L., Clokie M.R. (2022). Phage banks as potential tools to rapidly and cost-effectively manage antimicrobial resistance in the developing world. Curr. Opin. Virol..

[7] Hitchcock N.M., Nunes D.D.G., Shiach J., Hodel K.V.S., Barbosa J.D.V., Rodrigues L.A.P., Coler B.S., Soares M.B.P., Badaró R. (2023). Current Clinical Landscape and Global Potential of Bacteriophage Therapy. Viruses.

[8] Kulpakko J., Juusti V., Rannikko A., Hänninen P.E. (2022). Detecting disease associated biomarkers by luminescence modulating phages. Sci. Rep..

[9] Sundell K., Landor L., Castillo D., Middelboe M., Wiklund T. (2020). Bacteriophages as Biocontrol Agents for *Flavobacterium psychrophilum* Biofilms and Rainbow Trout Infections. Phage.

[10] Kim S.H., Adeyemi D.E., Park M.K. (2021). Characterization of a new and efficient polyvalent phage infecting *E. coli* o157:H7, *salmonella* spp., and *shigella sonnei*. Microorganisms.

[11] Martins W.M.B.S., Li M., Sands K., Lenzi M.H., Portal E., Mathias J., Dantas P.P., Migliavacca R., Hunter J.R., Medeiros E.A. (2022). Effective phage cocktail to combat the rising incidence of extensively drug-resistant *Klebsiella pneumoniae* sequence type 16. Emerg. Microbes Infect..

[12] Mapes A.C., Trautner B.W., Liao K.S., Ramig R.F. (2016). Development of expanded host range phage active on biofilms of multi-drug resistant *Pseudomonas aeruginosa*. Bacteriophage.

[13] Singla S., Harjai K., Katare O.P., Chhibber S. (2016). Encapsulation of Bacteriophage in Liposome Accentuates Its Entry in to Macrophage and Shields It from Neutralizing Antibodies. PLoS ONE.

[14] Hankin M.E. (2011). *The bactericidal action of the waters of the Jamuna and Ganges rivers on Cholera microbes*. Ann. Inst. Pasteur 10:511–523 (1896). Bacteriophage.

[15] Schullian D.M., Rogers F.B. (1979). Notes and Events. J. Hist. Med. Allied Sci..

[16] Twort F. (1915). An investigation on the nature of ultra-microscopic viruses. Lancet.

[17] Service P. (2007). On an invisible microbe antagonistic toward dysenteric bacilli: Brief note by Mr. F. D’Herelle, presented by Mr. Roux. Res. Microbiol..

[18] Moineau S. (2013). Bacteriophage.

[19] Zrelovs N., Dislers A., Kazaks A. (2020). Motley Crew: Overview of the Currently Available Phage Diversity. Front. Microbiol..

[20] E Bradley D. (1967). Ultrastructure of bacteriophage and bacteriocins. Bacteriol. Rev..

[21] Ackermann H. (1998). Tailed Bacteriophages: The Order Caudovirales. Adv. Virus Res..

[22] Ackermann H.W. (2006). Classification of bacteriophages. Bacteriophages.

[23] Ackermann H.-W. (2003). Bacteriophage observations and evolution. Res. Microbiol..

[24] Sieiro C., Areal-Hermida L., Pichardo-Gallardo Á., Almuiña-González R., De Miguel T., Sánchez S., Sánchez-Pérez Á., Villa T.G. (2020). A Hundred Years of Bacteriophages: Can Phages Replace Antibiotics in Agriculture and Aquaculture?. Antibiotics.

[25] Le S., He X., Tan Y., Huang G., Zhang L., Lux R., Shi W., Hu F. (2013). Mapping the Tail Fiber as the Receptor Binding Protein Responsible for Differential Host Specificity of *Pseudomonas aeruginosa* Bacteriophages PaP1 and JG004. PLoS ONE.

[26] Sun Y., Roznowski A.P., Tokuda J.M., Klose T., Mauney A., Pollack L., Fane B.A., Rossmann M.G. (2017). Structural changes of tailless bacteriophage ΦX174 during penetration of bacterial cell walls. Proc. Natl. Acad. Sci. USA.

[27] Storms Z.J., Sauvageau D. (2015). Modeling tailed bacteriophage adsorption: Insight into mechanisms. Virology.

[28] Bertozzi Silva J., Storms Z., Sauvageau D. (2016). Host receptors for bacteriophage adsorption. FEMS Microbiol. Lett..

[29] Rakhuba D.V., Kolomiets E.I., Dey E.S., Novik G.I. (2010). Bacteriophage Receptors, Mechanisms of Phage Adsorption and Penetration into Host Cell. Pol. J. Microbiol..

[30] Peng C., Hanawa T., Azam A.H., LeBlanc C., Ung P., Matsuda T., Onishi H., Miyanaga K., Tanji Y. (2019). Silviavirus phage ΦMR003 displays a broad host range against methicillin-resistant *Staphylococcus aureus* of human origin. Appl. Microbiol. Biotechnol..

[31] Azam A.H., Hoshiga F., Takeuchi I., Miyanaga K., Tanji Y. (2018). Analysis of phage resistance in *Staphylococcus aureus* SA003 reveals different binding mechanisms for the closely related Twort-like phages ΦSA012 and ΦSA039. Appl. Microbiol. Biotechnol..

[32] Chatterjee A., Johnson C.N., Luong P., Hullahalli K., McBride S.W., Schubert A.M., Palmer K.L., Carlson P.E., Duerkop B.A. (2019). Bacteriophage Resistance Alters Antibiotic-Mediated Intestinal Expansion of Enterococci. Infect. Immun..

[33] Ahamed S.T., Roy B., Basu U., Dutta S., Ghosh A.N., Bandyopadhyay B., Giri N. (2019). Genomic and Proteomic Characterizations of Sfin-1, a Novel Lytic Phage Infecting Multidrug-Resistant *Shigella* spp. and *Escherichia coli* C. Front. Microbiol..

[34] Altamirano F.G., Forsyth J.H., Patwa R., Kostoulias X., Trim M., Subedi V.O.R.I.P., Archer S., Morris V.O.R.I.P.C., Oliveira C., Kielty L. (2020). Bacteriophages targeting *Acinetobacter baumannii* capsule induce antimicrobial resensitization. bioRxiv.

[35] Chan B., Stanley G.L., Kortright K.E., Modak M., Ott I.M., Sun Y., Würstle S., Grun C., Kazmierczak B., Rajagopalan G. (2023). Personalized Inhaled Bacteriophage Therapy Decreases Multidrug-Resistant *Pseudomonas aeruginosa*. medRxiv.

[36] Hesse S., Rajaure M., Wall E., Johnson J., Bliskovsky V., Gottesman S., Adhya S. (2020). Phage Resistance in Multidrug-Resistant *Klebsiella pneumoniae* ST258 Evolves via Diverse Mutations That Culminate in Impaired Adsorption. mBio.

[37] Abedon S., Thomas-Abedon C. (2010). Phage Therapy Pharmacology. Curr. Pharm. Biotechnol..

[38] Kutter E., Raya R., Carlson K. (2005). Molecular Mechanisms of Phage Infection. Bacteriophages: Biology and Application.

[39] Little J.W. (2014). Lysogeny, Prophage Induction, and Lysogenic Conversion. Phages.

[40] Skorb E.V., Andreeva D.V., Raiski A.P., Belyasova N.A., Möhwald H., Sviridov D.V. (2011). Titanium dioxide-assisted photocatalytic induction of prophages to lytic cycle. Photochem. Photobiol. Sci..

[41] Moons P., Faster D., Aertsen A. (2013). Lysogenic Conversion and Phage Resistance Development in Phage Exposed *Escherichia coli* Biofilms. Viruses.

[42] Edgar R., Friedman N., Molshanski-Mor S., Qimron U. (2012). Reversing Bacterial Resistance to Antibiotics by Phage-Mediated Delivery of Dominant Sensitive Genes. Appl. Environ. Microbiol..

[43] Park J.Y., Moon B.Y., Park J.W., Thornton J.A., Park Y.H., Seo K.S. (2017). Genetic engineering of a temperate phage-based delivery system for CRISPR/Cas9 antimicrobials against *Staphylococcus aureus*. Sci. Rep..

[44] Yu P., Mathieu J., Li M., Dai Z., Alvarez P.J.J. (2016). Isolation of Polyvalent Bacteriophages by Sequential Multiple-Host Approaches. Appl. Environ. Microbiol..

[45] Duc H.M., Son H.M., Yi H.P.S., Sato J., Ngan P.H., Masuda Y., Honjoh K.-I., Miyamoto T. (2020). Isolation, characterization and application of a polyvalent phage capable of controlling *Salmonella* and *Escherichia coli* O157:H7 in different food matrices. Food Res. Int..

[46] Sui B., Han L., Ren H., Liu W., Zhang C. (2021). A Novel Polyvalent Bacteriophage vB_EcoM_swi3 Infects Pathogenic *Escherichia coli* and *Salmonella enteritidis*. Front. Microbiol..

[47] Tao C., Yi Z., Zhang Y., Wang Y., Zhu H., Afayibo D.J.A., Li T., Tian M., Qi J., Ding C. (2021). Characterization of a Broad-Host-Range Lytic Phage SHWT1 against Multidrug-Resistant *Salmonella* and Evaluation of Its Therapeutic Efficacy in vitro and in vivo. Front. Vet. Sci..

[48] Xu J., Chen M., He L., Zhang S., Ding T., Yao H., Lu C., Zhang W. (2016). Isolation and characterization of a T4-like phage with a relatively wide host range within *Escherichia coli*. J. Basic Microbiol..

[49] De Melo A.C.C., da Mata Gomes A., Melo F.L., Ardisson-Araújo D.M.P., De Vargas A.P.C., Ely V.L., Kitajima E.W., Ribeiro B.M., Wolff J.L.C. (2019). Characterization of a bacteriophage with broad host range against strains of *Pseudomonas aeruginosa* isolated from domestic animals. BMC Microbiol..

[50] Ackermann H.W., DuBow M.S. (1987). Viruses of Prokaryotes: General Properties of Bacteriophages.

[51] Hamdi S., Rousseau G.M., Labrie S.J., Tremblay D.M., Kourda R.S., Slama K.B., Moineau S. (2017). Characterization of two polyvalent phages infecting Enterobacteriaceae. Sci. Rep..

[52] Gupta R., Prasad Y. (2011). Efficacy of polyvalent bacteriophage P-27/HP to control multidrug resistant *staphylococcus aureus* associated with human infections. Curr. Microbiol..

[53] Hyman P. (2019). Phages for Phage Therapy: Isolation, Characterization, and Host Range Breadth. Pharmaceuticals.

[54] Auad L., Holgado A.D.R., Forsman P., Alatossava T., Raya R. (1997). Isolation and Characterization of a New *Lactobacillus delbrueckii* ssp. *bulgaricus* Temperate Bacteriophage. J. Dairy Sci..

[55] Lu Z., Breidt F., Fleming H., Altermann E., Klaenhammer T. (2003). Isolation and characterization of a *Lactobacillus plantarum* bacteriophage, ΦJL-1, from a cucumber fermentation. Int. J. Food Microbiol..

[56] Lin L., Han J., Ji X., Hong W., Huang L., Wei Y. (2011). Isolation and characterization of a new bacteriophage MMP17 from Meiothermus. Extremophiles.

[57] Liu C.-K., Liu C.-P., Leung C.-H., Sun F.-J. (2011). Clinical and microbiological analysis of adult perianal abscess. J. Microbiol. Immunol. Infect..

[58] Kong H., Yu F., Zhang W., Li X. (2017). Clinical and microbiological characteristics of pyogenic liver abscess in a tertiary hospital in East China. Medicine.

[59] Lin Y.-T., Cheng Y.-H., Chuang C., Chou S.-H., Liu W.-H., Yang T.-C., Kreiswirth B.N., Chen L. (2020). Molecular and Clinical Characterization of Multidrug-Resistant and Hypervirulent *Klebsiella pneumoniae* Strains from Liver Abscess in Taiwan. Antimicrob. Agents Chemother..

[60] Law N., Logan C., Yung G., Furr C.-L.L., Lehman S.M., Morales S., Rosas F., Gaidamaka A., Bilinsky I., Grint P. (2019). Successful adjunctive use of bacteriophage therapy for treatment of multidrug-resistant *Pseudomonas aeruginosa* infection in a cystic fibrosis patient. Infection.

[61] Ngiam L., Schembri M.A., Weynberg K., Guo J. (2021). Bacteriophage isolated from non-target bacteria demonstrates broad host range infectivity against multidrug-resistant bacteria. Environ. Microbiol..

[62] Feng T., Leptihn S., Dong K., Loh B., Zhang Y., Stefan M.I., Li M., Guo X., Cui Z. (2021). JD419, a *Staphylococcus aureus* Phage with a Unique Morphology and Broad Host Range. Front. Microbiol..

[63] Morello E., Saussereau E., Maura D., Huerre M., Touqui L., Debarbieux L. (2011). Pulmonary Bacteriophage Therapy on *Pseudomonas aeruginosa* Cystic Fibrosis Strains: First Steps towards Treatment and Prevention. PLoS ONE.

[64] Khorshidtalab M., Durukan I., Tufekci E.F., Nas S.S., Abdurrahman M.A., Kilic A.O. (2022). Isolation and Characterization of Lytic Bacteriophages from Wastewater with Phage Therapy Potentials against Gram-Negative Bacteria. Eurasian J. Med..

[65] Bhetwal A., Maharjan A., Shakya S., Satyal D., Ghimire S., Khanal P.R., Parajuli N.P. (2017). Isolation of Potential Phages against Multidrug-Resistant Bacterial Isolates: Promising Agents in the Rivers of Kathmandu, Nepal. BioMed Res. Int..

[66] Summers W.C. (1999). Felix d’Herelle and the Origins of Molecular Biology.

[67] Schultz E.W. (1929). The Bacteriophage as a Therapeutic Agent. Calif. West. Med..

[68] Ligon B. (2004). Penicillin: Its discovery and early development. Semin. Pediatr. Infect. Dis..

[69] Girija A.S., Priyadharsini J.V. (2019). CLSI based antibiogram profile and the detection of MDR and XDR strains of *Acinetobacter baumannii* isolated from urine samples. Med. J. Islam. Repub. Iran MJIRI.

[70] Shahkarami F., Rashki A., Ghalehnoo Z.R. (2014). Microbial Susceptibility and Plasmid Profiles of Methicillin-Resistant *Staphylococcus aureus* and Methicillin-Susceptible *S. aureus*. Jundishapur J. Microbiol..

[71] Gastmeier P., Schröder C., Behnke M., Meyer E., Geffers C. (2014). Dramatic increase in vancomycin-resistant enterococci in Germany. J. Antimicrob. Chemother..

[72] Kutter E.M., Kuhl S.J., Abedon S.T. (2015). Re-establishing a place for phage therapy in western medicine. Future Microbiol..

[73] Oduor J.M.O., Onkoba N., Maloba F., Nyachieo A. (2016). Experimental phage therapy against haematogenous multi-drug resistant *Staphylococcus aureus* pneumonia in mice. Afr. J. Lab. Med..

[74] Abedon S.T., Kuhl S.J., Blasdel B.G., Kutter E.M. (2011). Phage treatment of human infections. Bacteriophage.

[75] Borysowski J., Gorski A. (2008). Is phage therapy acceptable in the immunocompromised host?. Int. J. Infect. Dis..

[76] Liu D., Van Belleghem J.D., de Vries C.R., Burgener E., Chen Q., Manasherob R., Aronson J.R., Amanatullah D.F., Tamma P.D., Suh G.A. (2021). The Safety and Toxicity of Phage Therapy: A Review of Animal and Clinical Studies. Viruses.

[77] Jault P., Leclerc T., Jennes S., Pirnay J.P., Que Y.-A.A., Resch G., Rousseau A.F., Ravat F., Carsin H., Le Floch R. (2019). Efficacy and tolerability of a cocktail of bacteriophages to treat burn wounds infected by *Pseudomonas aeruginosa* (PhagoBurn): A randomised, controlled, double-blind phase 1/2 trial. Lancet Infect. Dis..

[78] Dedrick R.M., Guerrero-Bustamante C.A., Garlena R.A., Russell D.A., Ford K., Harris K., Gilmour K.C., Soothill J., Jacobs-Sera D., Schooley R.T. (2019). Engineered bacteriophages for treatment of a patient with a disseminated drug-resistant *Mycobacterium abscessus*. Nat. Med..

[79] Rhoads D.D., Wolcott R.D., Kuskowski M.A., Wolcott B.M., Ward L.S., Sulakvelidze A. (2009). Bacteriophage therapy of venous leg ulcers in humans: Results of a phase I safety trial. J. Wound Care.

[80] Wright A., Hawkins C.H., Änggård E.E., Harper D.R. (2009). A controlled clinical trial of a therapeutic bacteriophage preparation in chronic otitis due to antibiotic-resistant *Pseudomonas aeruginosa*; a preliminary report of efficacy. Clin. Otolaryngol. Allied Sci..

[81] Leitner L., Sybesma W., Chanishvili N., Goderdzishvili M., Chkhotua A., Ujmajuridze A., Schneider M.P., Sartori A., Mehnert U., Bachmann L.M. (2017). Bacteriophages for treating urinary tract infections in patients undergoing transurethral resection of the prostate: A randomized, placebo-controlled, double-blind clinical trial. BMC Urol..

[82] Alam Sarker S., Berger B., Deng Y., Kieser S., Foata F., Moine D., Descombes P., Sultana S., Huq S., Bardhan P.K. (2017). Oral application of *Escherichia coli* bacteriophage: Safety tests in healthy and diarrheal children from Bangladesh. Environ. Microbiol..

[83] Jiang L., Tan J., Hao Y., Wang Q., Yan X., Wang D., Tuo L., Wei Z., Huang G. (2020). Isolation and Characterization of a Novel Myophage Abp9 against Pandrug Resistant *Acinetobacater baumannii*. Front. Microbiol..

[84] Fish R., Kutter E., Wheat G., Blasdel B., Kutateladze M., Kuhl S. (2016). Bacteriophage treatment of intransigent diabetic toe ulcers: A case series. J. Wound Care.

[85] Khan F.M., Gondil V.S., Li C., Jiang M., Li J., Yu J., Wei H., Yang H. (2021). A Novel *Acinetobacter baumannii* Bacteriophage Endolysin LysAB54 with High Antibacterial Activity against Multiple Gram-Negative Microbes. Front. Cell. Infect. Microbiol..

[86] Wu M., Hu K., Xie Y., Liu Y., Mu D., Guo H., Zhang Z., Zhang Y., Chang D., Shi Y. (2019). A Novel Phage PD-6A3, and Its Endolysin Ply6A3, with Extended Lytic Activity against *Acinetobacter baumannii*. Front. Microbiol..

[87] Pallavali R.R., Degati V.L., Narala V.R., Velpula K.K., Yenugu S., Durbaka V.R.P. (2021). Lytic Bacteriophages against Bacterial Biofilms Formed by Multidrug-Resistant *Pseudomonas aeruginosa*, *Escherichia coli*, *Klebsiella pneumoniae*, and *Staphylococcus aureus* Isolated from Burn Wounds. Ther. Appl. Res..

[88] Drulis-Kawa Z., Weber-Dabrowska B., Łusiak-Szelachowska M., Doroszkiewicz W. (2005). Potential possibilities of using phage typing in elimination of multidrug resistant staphylococci. Pol. J. Microbiol..

[89] Mohammed M. (2017). Phage typing or CRISPR typing for epidemiological surveillance of *Salmonella Typhimurium*?. BMC Res. Notes.

[90] Velayudham A., Manavalan J., Vadivel S., Kaliyamoorthy B., Kuthalaramalingam S. (2016). Bacteriophage Typing of Methicillin Resistant *Staphylococcus aureus* and Changing Trend in their Antibiotic Profile. Ann. Int. Med. Dent. Res..

[91] Venturini C., Bowring B., Partridge S.R., Ben Zakour N.L., Fajardo-Lubian A., Ayala A.L., Qin J., Totsika M., van Galen G., Norris J. (2022). Co-Occurrence of Multidrug Resistant *Klebsiella pneumoniae* Pathogenic Clones of Human Relevance in an Equine Pneumonia Case. Microbiol. Spectr..

[92] Perera M.N., Abuladze T., Li M., Woolston J., Sulakvelidze A. (2015). Bacteriophage cocktail significantly reduces or eliminates *Listeria monocytogenes* contamination on lettuce, apples, cheese, smoked salmon and frozen foods. Food Microbiol..

[93] Kazi M., Annapure U.S. (2016). Bacteriophage biocontrol of foodborne pathogens. J. Food Sci. Technol..

[94] Amarillas L., Rubí-Rangel L., Chaidez C., González-Robles A., Lightbourn-Rojas L., León-Félix J. (2017). Isolation and Characterization of phiLLS, a Novel Phage with Potential Biocontrol Agent against Multidrug-Resistant *Escherichia coli*. Front. Microbiol..

[95] Tian L., He L., Jackson K., Saif A., Khan S., Wan Z., Didar T.F., Hosseinidoust Z. (2022). Self-assembling nanofibrous bacteriophage microgels as sprayable antimicrobials targeting multidrug-resistant bacteria. Nat. Commun..

[96] Le T.S., Southgate P.C., O’connor W., Poole S., Kurtböke D.I. (2018). Bacteriophages as Biological Control Agents of Enteric Bacteria Contaminating Edible Oysters. Curr. Microbiol..

[97] Bao J., Wu N., Zeng Y., Chen L., Li L., Yang L., Zhang Y., Guo M., Li L., Li J. (2020). Non-active antibiotic and bacteriophage synergism to successfully treat recurrent urinary tract infection caused by extensively drug-resistant *Klebsiella pneumoniae*. Emerg. Microbes Infect..

[98] Arumugam S.N., Rudraradhya A.C., Sadagopan S., Sukumaran S., Sambasivam G., Ramesh N. (2018). Analysis of Susceptibility Patterns of *Pseudomonas aeruginosa* and Isolation, Characterization of Lytic Bacteriophages Targeting Multi Drug Resistant *Pseudomonas aeruginosa*. Biomed. Pharmacol. J..

[99] Popova A.V., Zhilenkov E.L., Myakinina V.P., Krasilnikova V.M., Volozhantsev N.V. (2012). Isolation and characterization of wide host range lytic bacteriophage AP22 infecting *Acinetobacter baumannii*. FEMS Microbiol. Lett..

[100] Nyachieo A., Alafi S., Mutai I.J., Ngolobe B., Nabunje R., Nakavuma J.L. (2021). Isolation and Characterization of Novel Lytic Phages to Combat Multidrug-Resistant *E. coli* and *Salmonella* spp. J. Microbiol. Infect. Dis..

[101] Arumugam S.N., Manohar P., Sukumaran S., Sadagopan S., Loh B., Leptihn S., Nachimuthu R. (2022). Antibacterial efficacy of lytic phages against multidrug-resistant *Pseudomonas aeruginosa* infections in bacteraemia mice models. BMC Microbiol..

[102] Dhungana G., Nepal R., Regmi M., Malla R. (2021). Pharmacokinetics and Pharmacodynamics of a Novel Virulent *Klebsiella* Phage Kp_Pokalde_002 in a Mouse Model. Front. Cell. Infect. Microbiol..

[103] Tang S.-S., Biswas S.K., Tan W.S., Saha A.K., Leo B.-F. (2019). Efficacy and potential of phage therapy against multidrug resistant *Shigella* spp. PeerJ.

[104] Gu J., Liu X., Li Y., Han W., Lei L., Yang Y., Zhao H., Gao Y., Song J., Lu R. (2012). A Method for Generation Phage Cocktail with Great Therapeutic Potential. PLoS ONE.

[105] Shahin K., Bouzari M., Komijani M., Wang R. (2020). A New Phage Cocktail against Multidrug, ESBL-Producer Isolates of *Shigella sonnei* and *Shigella flexneri* with Highly Efficient Bacteriolytic Activity. Microb. Drug Resist..

[106] Pereira S., Santos L., Klumpp J., Almeida A. (2016). Potential of phage cocktails in the inactivation of *Enterobacter cloacae*—An in vitro study in a buffer solution and in urine samples. Virus Res..

[107] Haines M.E.K., Hodges F.E., Nale J.Y., Mahony J., van Sinderen D., Kaczorowska J., Alrashid B., Akter M., Brown N., Sauvageau D. (2021). Analysis of Selection Methods to Develop Novel Phage Therapy Cocktails against Antimicrobial Resistant Clinical Isolates of Bacteria. Front. Microbiol..

[108] Lin T.-Y., Lo Y.-H., Tseng P.-W., Chang S.-F., Lin Y.-T., Chen T.-S. (2012). A T3 and T7 Recombinant Phage Acquires Efficient Adsorption and a Broader Host Range. PLoS ONE.

[109] Yehl K., Lemire S., Yang A.C., Ando H., Mimee M., Torres M.D.T., de la Fuente-Nunez C., Lu T.K. (2019). Engineering Phage Host-Range and Suppressing Bacterial Resistance through Phage Tail Fiber Mutagenesis. Cell.

[110] Dong X., Pan P., Zheng D.W., Bao P., Zeng X., Zhang X.Z. (2020). Bioinorganic hybrid bacteriophage for modulation of intestinal microbiota to remodel tumor-immune microenvironment against colorectal cancer. Sci. Adv..

[111] He X., Yang Y., Guo Y., Lu S., Du Y., Li J.-J., Zhang X., Leung N.L.C., Zhao Z., Niu G. (2020). Phage-Guided Targeting, Discriminative Imaging, and Synergistic Killing of Bacteria by AIE Bioconjugates. J. Am. Chem. Soc..

[112] Dong S., Shi H., Zhang X., Chen X., Cao D., Mao C., Gao X., Wang L. (2018). Difunctional bacteriophage conjugated with photosensitizers for *Candida albicans*-targeting photodynamic inactivation. Int. J. Nanomed..

[113] Anany H., Chen W., Pelton R., Griffiths M.W. (2011). Biocontrol of *Listeria monocytogenes* and *Escherichia coli* O157:H7 in Meat by Using Phages Immobilized on Modified Cellulose Membranes. Appl. Environ. Microbiol..

[114] Abdelsattar A.S., Nofal R., Makky S., Safwat A., Taha A., El-Shibiny A. (2021). The Synergistic Effect of Biosynthesized Silver Nanoparticles and Phage ZCSE2 as a Novel Approach to Combat Multidrug-Resistant *Salmonella enterica*. Antibiotics.

[115] Lopes A., Pereira C., Almeida A. (2018). Sequential Combined Effect of Phages and Antibiotics on the Inactivation of *Escherichia coli*. Microorganisms.

[116] Mai V., Ukhanova M., Reinhard M.K., Li M., Sulakvelidze A. (2015). Bacteriophage administration significantly reduces *Shigella* colonization and shedding by *Shigella*-challenged mice without deleterious side effects and distortions in the gut microbiota. Bacteriophage.

[117] Llanos-Chea A., Citorik R.J., Nickerson K.P., Ingano L., Serena G., Senger S., Lu T.K., Fasano A., Faherty C.S. (2019). Bacteriophage Therapy Testing against *Shigella flexneri* in a Novel Human Intestinal Organoid-Derived Infection Model. J. Pediatr. Gastroenterol. Nutr..

